# Structure–Antioxidant–Antiproliferative Activity Relationships of Natural C7 and C7–C8 Hydroxylated Flavones and Flavanones

**DOI:** 10.3390/antiox8070210

**Published:** 2019-07-07

**Authors:** Sandra Sordon, Jarosław Popłoński, Magdalena Milczarek, Martyna Stachowicz, Tomasz Tronina, Alicja Z. Kucharska, Joanna Wietrzyk, Ewa Huszcza

**Affiliations:** 1 Department of Chemistry, Wrocław University of Environmental and Life Sciences, Norwida 25, 50-375 Wrocław, Poland; 2 Department of Experimental Oncology, Hirszfeld Institute of Immunology and Experimental Therapy, Polish Academy of Sciences, Weigla 12, 53-114 Wrocław, Poland; 3 Department of Fruit, Vegetable and Plant Nutraceutical Technology, Wrocław University of Environmental and Life Sciences, Chełmońskiego 37, 51-630 Wrocław, Poland

**Keywords:** flavones, flavanones, hydroxylation, catechol moiety, antioxidant activity, anticancer activity

## Abstract

Common food flavonoids: chrysin, apigenin, luteolin, diosmetin, pinocembrin, naringenin, eriodictyol, hesperetin, and their analogues with an additional hydroxyl group at the C-8 position obtained via biotransformation were tested for antioxidant activity using the ABTS, DPPH, and ferric ion reducing antioxidant power (FRAP) methods. They were also tested for antiproliferative activity against selected human cancer cell lines—MV-4-11 (biphenotypic B myelomonocytic leukemia), MCF7 (breast carcinoma), LoVo (colon cancer), LoVo/DX (colon cancer doxorubicin resistant), and DU 145 (prostate cancer)—and two normal human cell lines—MCF 10A (breast cells) and HLMEC (lung microvascular endothelial cells). Flavonoids with a C7–C8 catechol moiety indicated much higher antioxidant activity compared with the C7 hydroxy analogues. However, because they were unstable under the assay conditions, they did not show antiproliferative activity or it was very low.

## 1. Introduction

Flavonoids belong to a large group of polyphenolic compounds that are characterized by the structure of benzo-γ-pyrone, the most common and widely distributed plant secondary metabolite. They exert a considerable influence on the growth and development of plants, protect them from UV radiation and bacterial and fungal infections, and provide color to fruits and flowers [1].

Flavonoids present in food have beneficial effects on human health. It is well known that these compounds exhibit a broad spectrum of biological activities, for example, anti-inflammatory, anticancer, antimicrobial, antiviral, antidiabetic, cardioprotective, and estrogenic [2]. Most of the prohealth activities of flavonoid compounds are associated with their antioxidant abilities. Especially due to these properties, flavonoids are considered to be useful in preventing and/or treating diseases such as cancers [3]. One of the factors causing the development of tumors is increased production of reactive oxygen species and the accompanying intensification of oxidative modifications of lipids, proteins, and nucleic acids. In this regard, flavonoids are responsible for inhibiting the formation of reactive oxygen species [4]. Apart from antioxidant activity, these compounds play an important role in many others mechanisms of action for prevention against cancer, including carcinogen inactivation, antiproliferation, cell cycle arrest, induction of apoptosis, inhibition of angiogenesis, reversal of multidrug resistance, or a combination of these mechanisms [5]. Despite numerous studies, the antitumor action mechanism of flavonoid compounds is still not very clear. There are many reports on the relationship between the structure of flavonoid molecules and their antioxidant activity [5,6,7], including the ability to scavenge free radicals or to reduce iron ions. It is considered that a large number of hydroxyl groups in the flavonoid molecule results in much higher antioxidant activity [8,9,10,11]. The catechol group present in the B-ring is most often mentioned as the crucial element in the molecule responsible for its high antioxidant activity [12]. It is also believed that flavonoids with a catechol moiety in ring A may exhibit significant antioxidant action [13]. The presence of the 2,3-double bond, as well as the appropriate number and positions of hydroxyl groups, particularly the *ortho*-hydroxyl system in the B-ring, are also characterized as potent for tumor inhibition [14,15].

Chemical synthesis of flavonoid derivatives with a catechol moiety is challenging and requires many protection–deprotection steps. Application of microorganisms as a biocatalyst is a relevant strategy for performing the *ortho*-hydroxylation reaction, thus directly producing the desired structures in a single-step reaction. Additionally, biocatalysts characterize high regio- and stereoselectivity [16].

There are few reports regarding the antioxidant and anticancer activity of flavonoids with hydroxyl groups in the *ortho* position in the A-ring, which is probably associated with the difficulties of isolating and synthesizing this type of compound. Therefore, the aim of this study was a comparative analysis of the antioxidant and antiproliferative activity of common food natural flavones and flavanones and their analogues with the catechol C7–C8 moiety obtained by enzymatic regioselective *ortho*-hydroxylation of the A-ring.

## 2. Materials and Methods 

### 2.1. Compounds

**Figure 1:**
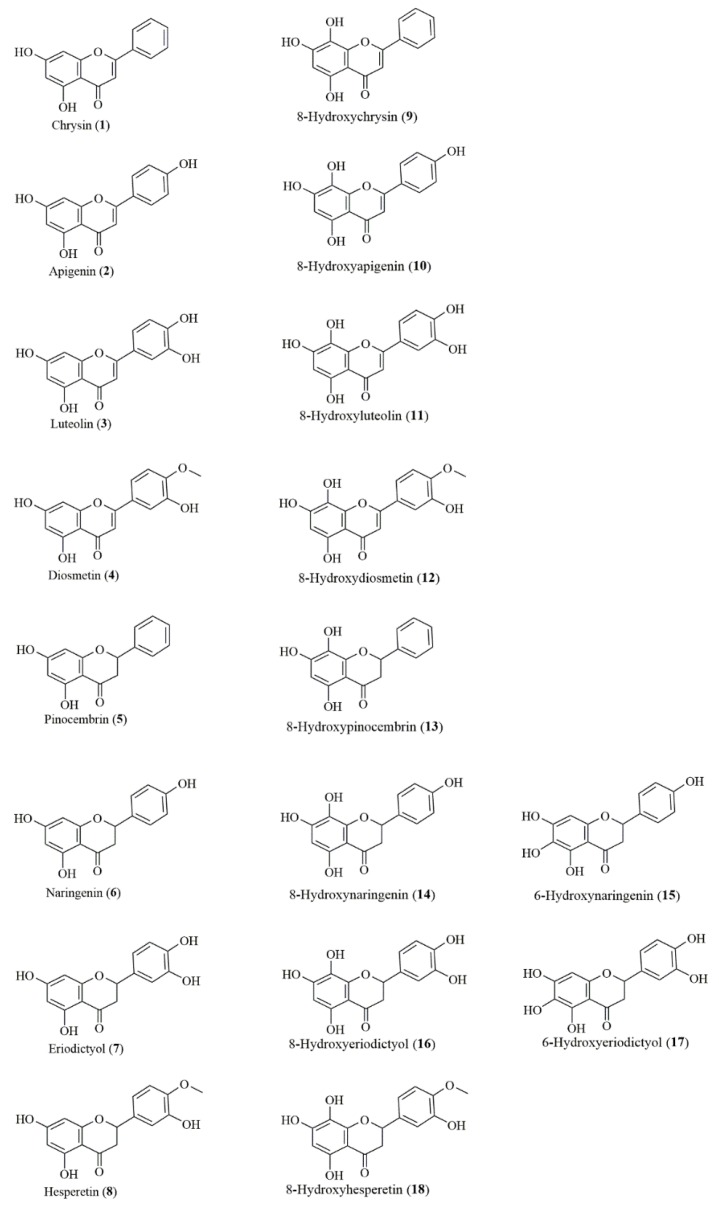
Chemical structures of tested flavonoids.

Figure 1 displays the structures of the studied natural flavonoids. 

Chrysin (**1**) and naringenin (**6**) were acquired from Sigma-Aldrich (St. Louis, MO, USA); hesperetin (**8**) from Alfa-Aesar (Thermo Fisher, Karlsruhe, Germany); and apigenin (**2**), luteolin (**3**), and diosmetin (**4**) from Carbosynth (Berkshire, UK). Pinocembrin (**5**) and eriodictyol (**7**) were obtained via chemical synthesis according to the method described by Sordon et al. [17].

Hydroxylated flavonoids at the C-8 position tested in this study were prepared via biotransformation according to our previously published procedure concerning regioselective *ortho*-hydroxylation of natural flavonoids catalyzed by the yeast *Rhodotorula glutinis* KCh735 [18]. This research extends the portfolio of possible routes to C7–C8 flavonoids and describes the method of production of 8-hydroxychrysin (**9**), 8-hydroxyapigenin (**10**), 8-hydroxyluteolin (**11**), 8-hydroxyhesperetin (**18**), and the mixture of 8-hydroxynaringenin (**14**) and 6-hydroxynaringenin (**15**).

8-Hydroxydiosmetin (**12**), 8-hydroxypinocembrin (**13**), and the mixture of 8-hydroxyeriodictyol (**16**) and 6-hydroxyeriodictyol (**17**) were obtained and purified in the analogous method [18]. 

8-Hydroxydiosmetin (**12**): ^1^H NMR (600 MHz, DMSO-*d_6_*) δ (ppm): 3.85 (3H, s, C4’-OCH_3_), 6.26 (1H, s, H-6), 6.70 (1H, s, H-3), 7.08 (1H, d, J = 8.5 Hz, H-5’), 7.52 (1H, d, J = 2.3 Hz, H-2’), 7.61 (1H, dd, J = 2.3 Hz; 8.5, H-6’), 8.75 (1H, s, 8-OH), 9.45 (1H, s, 3’-OH), 10.51 (1H, s, 7-OH), 12.35 (1H, s, 5-OH); ^13^C NMR (150 MHz, DMSO-*d_6_*) δ (ppm): 55.8 (C4’-OCH_3_), 98.6 (C-6), 103.0 (C-3), 103.4 (C-10), 112.1 (C-5’), 113.2 (C-2’), 118.9 (C-6’), 123.3 (C-1’), 125.1 (C-8), 145.5 (C-9), 146.7 (C-3’), 151.1 (C-4’), 153.0 (C-5), 153.4 (C-7), 163.4 (C-2), 182.1 (C-4). 

8-Hydroxypinocembrin (**13**): ^1^H NMR (600 MHz, DMSO-*d_6_*) δ (ppm): 2.83 (1H, dd, J = 3.1; 17.1 Hz, H-3_eq_), 3.23 (1H, dd, J = 12.2; 17.1 Hz, H-3_ax_), 5.58 (1H, dd, J = 3.1; 12.2 Hz, H-2), 5.96 (1H, s, H-6), 7.40 (1H, m, H-4’), 7.45 (2H, m, H-3’, H-5’), 7.58 (2H, m, H-2’, H-6’), 11.73 (1H, s, 5-OH); ^13^C NMR (150 MHz, DMSO-*d_6_*) δ (ppm): 42.4 (C-3), 78.4 (C-2), 95.6 (C-6), 101.7 (C-10), 125.7 (C-8), 126.7 (C-2’, C-6’), 128.4 (C-4’), 128.5 (C-3’, C-5’), 139.0 (C-1’), 149.1 (C-9), 155.8 (C-7), 156.6 (C-5), 196.2 (C-4). 

8-Hydroxyeriodictyol (**16**): ^1^H NMR (600 MHz, DMSO-*d_6_*) δ (ppm): 2.67 (1H, dd, J = 3.1; 17.1 Hz, H-3_eq_), 3.11 (1H, dd, J = 12.1; 17.1 Hz, H-3_ax_), 5.33 (1H, dd, J = 3.1; 12.1 Hz, H-2), 5.90 (1H, s, H-6), 6.74 (1H, m, H-5’), 6.76 (1H, dd, J = 2.1; 8.2 Hz, H-6’), 6.90 (1H, d, J = 2.1 Hz, H-2’), 8.05 (1H, s, 7-OH), 10.40 (1H, s, 8-OH), 11.73 (1H, s, 5-OH); ^13^C NMR (150 MHz, DMSO-*d_6_*) δ (ppm): 42.4 (C-3), 78.5 (C-2), 95.3 (C-6), 101.7 (C-10), 114.5 (C-2’), 115.3 (C-5’), 118.0 (C-6’), 125.6 (C-8), 129.7 (C-1’), 145.2 (C-4’), 145.6 (C-3’), 149.4 (C-9), 155.8 (C-7), 156.4 (C-5), 196.6 (C-4). 

6-Hydroxyeriodictyol (**17**): ^1^H NMR (600 MHz, DMSO-*d_6_*) δ (ppm): 2.63 (1H, dd, J = 3.0; 17.1 Hz, H-3_eq_), 3.14 (1H, dd, J = 12.6; 17.1 Hz, H-3_ax_), 5.28 (1H, dd, J = 3.0; 12.6 Hz, H-2), 5.91 (1H, s, H-8), 6.71 (2H, m, H-5’, H-6’), 6.84 (1H, m, H-2’), 8.18 (1H, s, 7-OH), 10.35 (1H, s, 6-OH), 11.96 (1H, s, 5-OH); ^13^C NMR (150 MHz, DMSO-*d_6_*) δ (ppm): 42.4 (C-3), 78.5 (C-2), 94.7 (C-8), 101.7 (C-10), 114.3 (C-2’), 115.3 (C-5’), 117.9 (C-6’), 126.3 (C-6), 129.8 (C-1’), 145.2 (C-4’), 145.6 (C-3’), 150.2 (C-9), 155.2 (C-5), 155.7 (C-7), 197.0 (C-4). 

### 2.2. Analytical Methods 

HPLC analysis were carried out using a Thermo Scientific Dionex Ultimate 3000 UHPLC+ instrument (Thermo Scientific, Waltham, MA, USA) with a photodiode array detector (detection in wavelength: 210–450 nm). A C-18 analytical column ZORBAX Eclipse XDB (5 µm, 4.6 × 250 mm, Agilent, Santa Clara, CA, USA) was used at the flow rate of 1 mL/min. Chromatographic separation was achieved using the isocratic elution of 50% A (0.05% formic acid water solution) and 50% B (methanol containing 0.05% of formic acid) for 2 min, then linear gradient of B from 50% to 95% for 10 min and isocratic elution of 95% B for 2 min.

The obtained products were identified using nuclear magnetic resonance (NMR) spectroscopy. ^1^H-NMR, ^13^C-NMR, ^1^H-^1^H-NMR (COSY), and ^1^H-^13^C-NMR (HSQC, HMBC) spectra analyses were recorded using a DRX Bruker Avance TM 600 (600 MHz) instrument.

### 2.3. Antioxidant Activity

The antioxidant capacity of flavonoids was measured in three separate tests: ABTS, DPPH, and ferric ion reducing antioxidant power (FRAP) assays (ABTS, DPPH, and FRAP reagents from Sigma-Aldrich). All tested compounds were dissolved in DMSO. All determinations were performed in triplicate. Standard curves were prepared for all assays using different concentrations of Trolox (6-hydroxy-2,5,7,8-tetramethylchroman-2-carboxylic acid) (Sigma-Aldrich). Trolox is a water-soluble analogue of vitamin E with high antioxidant activity, commonly used as a control and a reference in studies of antioxidant activity [19]. The results were expressed as the equivalent of μmol Trolox per μmol of the test compound. The absorbance was measured using a UV-2401 PC spectrophotometer (Shimadzu, Kyoto, Japan). Concentrations of the tested samples were in the range from 50 µg/mL to 10 mg/mL.

#### 2.3.1. ABTS Radical Cation Decolorization Assay 

The radical cation scavenging capacity of the tested compounds was examined against ABTS^+^ (2,2’-azino-bis(3-ethylbenzo-thiazoline-6-sulfonic acid) according to the procedure described by Re et al. [20] with some modifications. The test sample (30 μL) of known concentration was mixed in a polystyrene cuvette with 3 mL of ABTS solution with a measured absorption of 0.700 at a wavelength of λ = 734 nm. After 6 min of incubation, the absorption of samples was recorded. The ABTS reagent was freshly prepared before each experiment.

#### 2.3.2. DPPH Free Radical Scavenging Activity Assay

The DPPH (2,2-diphenyl-1-picrylhydrazyl) radical scavenging activity of samples was determined according to the method described by Yen and Chen [21] with some modifications. Samples (0.1 mL) of known concentration were mixed in polystyrene cuvettes with 2 mL of 0.04 mM DPPH in ethanol and 0.4 mL of ethanol. The absorbance was measured after 10 min at λ = 517 nm. The DPPH reagent was freshly prepared before each experiment.

#### 2.3.3. FRAP Assay

The FRAP method is based on measuring the ability of antioxidants to reduce ferric 2,4,6-tris(2-pyridyl)-1,3,5-triazine [Fe(III)-TPTZ] in the blue colored ferrous form, which absorbs light at λ = 593 nm [22]. The Fe(III)-TPTZ reagent was prepared as a mixture of acetate buffer (pH 3.6), 10 mM TPTZ in 40 mM HCl, and 20 mM FeCl_3_ in the ratio of 10:1:1 (v/v/v). The FRAP reagent was freshly prepared before each experiment. The samples (0.1 mL) were mixed in polystyrene cuvettes with 3 mL of ferric complex and 0.9 mL of distilled water. The absorbance was measured after 10 min at λ = 593 nm.

### 2.4. Antiproliferative Activity In Vitro

The antiproliferative studies were performed in vitro using human cancer cell lines—MV-4-11 (biphenotypic B myelomonocytic leukemia), MCF7 (breast carcinoma), LoVo (colon cancer), LoVo/DX (colon cancer doxorubicin-resistant), and DU 145 (prostate cancer)—and normal human cell lines—MCF 10A (breast cells) and HLMEC (lung microvascular endothelial cells). The cell lines MV-4-11, LoVo, DU 145, and MCF 10A were obtained from the American Type Culture Collection (Rockville, Maryland, USA), MCF7 from the European Collection of Authenticated Cell Cultures (ECACC, Salisbury, UK), LoVo/DX courtesy of Professor E. Borowski (Technical University of Gdansk, Poland), and HLMECs (which had been already immortalized by transfection with pSV3-neoplasmid containing large T-antigen gene) were the kind gift of Professor Claudine Kieda (Center for Molecular Biophysics, Orleans, France). The cell lines are being maintained in the Hirszfeld Institute of Immunology and Experimental Therapy, Polish Academy of Sciences (HIIET, PAS), Wroclaw, Poland. All cell lines were grown at 37 °C with 5% CO_2_ humidified atmosphere. The MV-4-11 cells were cultured in RPMI 1640 medium with GlutaMAX (Gibco, Scotland, UK), supplemented with 1 mM sodium pyruvate and 10% fetal bovine serum (both from Sigma-Aldrich, Poznan, Poland). The MCF7 cells were cultured in Eagle medium (HIIET, PAS, Wroclaw, Poland) supplemented with 2 mM L-glutamine, 10% fetal bovine serum, 1% nonessential amino acid solution, and 0.8 mg/L of insulin (all from Sigma-Aldrich, Poznan, Poland). The LoVo and LoVo/DX cells were cultured in a 1:1 (v/v) mixture of RPMI 1640 and Opti-MEM (both HIIET, PAS, Wroclaw, Poland), supplemented with 1 mM sodium pyruvate, 2 mM l-glutamine, and 5% fetal bovine serum (all from Sigma-Aldrich, Poznan, Poland). For LoVo/DX cells, additionally 10 µg/100 mL of doxorubicin (Accord Healthcare) was added to the medium. DU 145 cells were cultured in Eagle medium (HIIET, PAS, Wroclaw, Poland) supplemented with 1 mM sodium pyruvate, 4 mM l-glutamine, and 10% fetal bovine serum (all from Sigma-Aldrich, Poznan, Poland). The MCF 10A cells were cultured in the F-12 nutrient mixture (Gibco, Scotland, UK), supplemented with 5% horse serum (Gibco, Scotland, UK), 0.05 μg/mL of cholera toxin (*Vibrio cholerae*), 0.5 μg/mL of hydrocortisone, 10 μg/mL of insulin, and 20 ng/mL of human epidermal growth factor (all from Sigma-Aldrich, Poznan, Poland). The HLMEC cells were cultured in the RPMI 1640 medium (HIIET, PAS, Wroclaw, Poland) with the addition of 2 mM l-glutamine (Sigma-Aldrich, Poznan, Poland) and 10% fetal bovine serum (GE Healthcare). All culture media were supplemented with 100 units/mL penicillin (Polfa Tarchomin S.A., Warsaw, Poland) and 100 µg/mL streptomycin (Sigma-Aldrich, Poznan, Poland). Twenty-four hours before addition of the tested agents, the cells were plated in 96-well plates (Sarstedt, Germany) at a density of 1 × 10^4^ (1 × 10^3^ for HLMEC and 0.75 × 10^4^ for MCF7) cells per well in 100 μL of culture medium.

Tested compounds were dissolved in DMSO and then diluted in a 1:1 (v/v) mixture of RPMI 1640 (HIIET, PAS, Wroclaw, Poland) and Opti-MEM (Gibco, Scotland, UK) medium supplemented with 2 mM L-glutamine (Sigma-Aldrich, Poznan, Poland), 5% fetal bovine serum (GE Healthcare), 100 units/mL penicillin (Polfa Tarchomin S.A., Warsaw, Poland), and 100 µg/mL streptomycin (Sigma-Aldrich, Poznan, Poland). The antiproliferative effects of the flavonoids were examined after 72 h of exposure of the cultured cells to varying concentrations of the tested agents (from 0.1 to 100 μg/mL) using the MTT (for MV-4-11 cells) or SRB assay described below. Cisplatin (chemotherapy drug used to treat a number of cancers) was used as a positive control. The results are presented as an inhibitory concentration 50 (IC_50_), which is the concentration of the tested agent that inhibits proliferation of the cell population by 50%. IC_50_ values for each experiment were calculated separately. The mean values ± SD are shown in the Table 1.

#### 2.4.1. MTT Assay

After compound treatment, 20 µL of MTT (3-(4,5-dimethylthiazol-2-yl)-2,5-diphenyl tetrazolium bromide (Sigma-Aldrich, Poznan, Poland); stock solution: 5 mg/mL) solution was added to each well. Viable cell mitochondria reduce the pale yellow MTT to a navy-blue formazan. After 4 h of incubation at 37 °C, 80 µL of the lysing mixture was added to each well (lysing mixture: 225 mL N,N-dimethylformamide (Avantor Performance Materials, Gliwice, Poland), 67.5 g sodium dodecyl sulfate (Sigma-Aldrich, Poznan, Poland), and 275 mL distilled water). The optical densities of the samples were read on a Synergy H4 multimode microplate reader (BioTek Instruments, VT, USA) at wavelength λ = 570 nm. 

#### 2.4.2. SRB Assay

The cells were attached to the bottom of plastic wells by fixing them with cold 50% trichloroacetic acid (Sigma-Aldrich, Poznan, Poland) on the top of the culture medium in each well. The 96-well plates were incubated at room temperature for 1 h and after that washed five times with distilled water. The cellular material fixed with trichloroacetic acid was stained with 0.1% sulforhodamine B (Sigma-Aldrich, Poznan, Poland) dissolved in 1% acetic acid (POCH, Gliwice, Poland) for 30 min. Unbound dye was removed by rinsing (five times) in 1% acetic acid. The protein-bound dye was extracted with 10 mM unbuffered Tris base (POCH). The optical densities of the samples were read on a Synergy H4 multimode microplate reader (BioTek Instruments, VT, USA) at λ = 540 nm wavelength. 

### 2.5. Stability of Flavonoids under Antiproliferative Test Conditions

The tests were carried out on two corresponding pairs of compounds, with or without the catechol moiety at position C7, C8 or C6, C7. They were: naringenin (**6**) and the mixture of 8-hydroxynaringenin (**14**) and 6-hydroxynaringenin (**15**), as well as apigenin (**2**) and 8-hydroxyapigenin (**10**). One cell line sensitive to flavonoids with a catechol moiety (HLMEC) and one insensitive (MCF7) were used in these studies.

#### 2.5.1. Stability of Flavonoids before Administration to the Cell Cultures

Flavonoids were dissolved (as described in Section 2.4) to the concentration of 100 µg/mL. The solutions corresponding to the solutions before the administration to the cell cultures were incubated in 96-well plates for 3 h and transferred to microtubes containing 400 µL of cold methanol (−20 °C) and kept at −20 °C for 1 h. After centrifugation at 12,000× *g* for 10 min, samples were analyzed by HPLC. Control experiments were also carried out to determine the stability of flavonoids in DMSO under analogous conditions.

#### 2.5.2. Stability of Flavonoids in the Cell Cultures

Flavonoids dissolved (as described in Section 2.4) were added to the HLMEC and MCF7 line cultures, and the final concentration was 100 μg/mL. The cultures were incubated in 96-well plates for 3 h and transferred to microtubes containing 400 µL of cold methanol (−20 °C) and kept at −20 °C for 1 h. After centrifugation at 12,000× *g* for 10 min, samples were analyzed by HPLC. To determine the stability of flavonoids in HLMEC and MCF7 culture media (not containing cells) under analogous conditions, control experiments were also carried out.

## 3. Results

### 3.1. Biotransformation

Flavone **12** (8-hydroxydiosmetin) was the only product of the six-day biotransformation of diosmetin (**4**). It was isolated with the yield of 15%. 8-Hydroxypinocembrin (**13**) was obtained by seven days of enzymatic hydroxylation of pinocembrin (**5**) by *R. glutinis* (yield: 33%). Incubation of eriodictyol (**7**) with *R. glutinis* for three days resulted in two products: 8-hydroxyeriodictyol (**16**) and 6-hydroxyeriodictyol (**17**) with a yield of 8% and 11%, respectively. The purity of obtained biotransformation products was over 98% (according to HPLC and NMR methods). 

The chemical structures of this compounds were unambiguously identified by NMR. The ^1^H-NMR and ^13^C-NMR spectra of **12**, **13**, **16**, and **17** are available in the Supplementary Materials. The NMR data of obtained biotransformation products were in agreement with literature data [23,24,25] and clearly indicate that additional hydroxyl groups occurred at the C8 position (compounds **12**, **13**, and **16**) or the C6 position (compound **17**).

### 3.2. Antioxidant Activity

ABTS, DPPH, and FRAP assays are the most popular spectrometric methods used for determination of the antioxidant capacity of natural compounds. The ABTS and DPPH methods are based on the scavenging of radicals (2,2’-azino-bis(3-ethylbenzthiazoline-6-sulphonic acid and 2,2-diphenyl-1-picrylhydrazyl, respectively), whereas the FRAP method is based on the reduction of the ferric ion TPTZ (2,4,6-tri(2-pyridyl)-1,3,5-triazine) complex by the antioxidant compounds.

**Figure 2:**
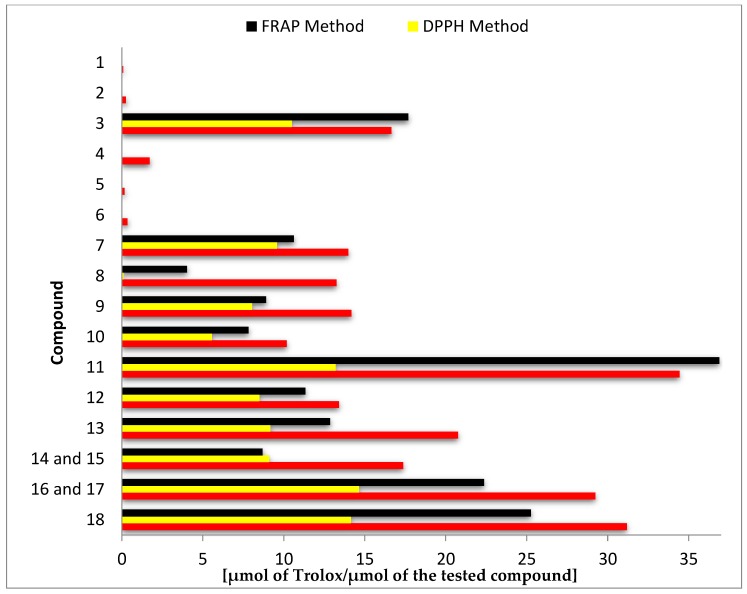
Antioxidant activity of tested compounds (**1**–**18**) determined in separate tests: ABTS, DPPH, and ferric ion reducing antioxidant power (FRAP).

Results of antioxidant activity assays using ABTS, DPPH, and FRAP expressed as μmol of Trolox per μmol of the test compound are summarized in Figure 2 and Table S1 (Supplementary Materials).

### 3.3. Antiproliferative Activity In Vitro

The results of antiproliferative activity of tested compounds are summarized in Table 1 presented as the IC_50_ values. 

## 4. Discussion

### 4.1. Biotransformation

Using a useful tool for synthesis of 8-hydroxy flavonoids, which is the yeast *R. glutinis* KCh735, we obtained a variety of C5-hydroxy flavones and flavanones with the C7–C8 catechol moiety in the ring A. These products of microbial transformation that occur in nature in small amounts, to the best of our knowledge, have not been widely studied so far, and together with the substrates used in biotransformation (compounds **1**–**8**), create a library of closely structurally related flavonoids, perfectly suited for studying structure–activity relationships (Figure 1).

### 4.2. Antioxidant Activity

The highest antioxidant activity in all three tests was demonstrated by the biotransformation products 8-hydroxyluteolin (**11**), 8-hydroxyhesperetin (**18**), and the mixture of 8- and 6-hydroxyeriodictyol (**16**, **17**). In the ABTS test, the mentioned compounds were more than twice as active as the substrates from which they were obtained, whereas in the DPPH and FRAP tests, the highest increase of activity was noted for the hesperetin (**8**) and 8-hydroxyhesperetin (**18**) pair (144 and 6 times higher, respectively) (Figure 2 and Table S1 of the Supplementary Materials).

Comparison of the antioxidant activity of other substrate–biotransformation product pairs, despite lower numerical values, indicated even more significant increases in activity. The best effects were achieved by hydroxylation of chrysin (**1**) to 8-hydroxychrysin (**9**) (increase of about 170-fold in ABTS test and about 2000-fold in DPPH test) and pinocembrin (**5**) to 8-hydroxypinocembrin (**12**) (increase of about 130-fold in ABTS test and about 1800-fold in DPPH test) (Figure 2 and Table S1 of the Supplementary Materials).

Flavones and flavanones with a C7–C8 catechol moiety in the ring A exhibit much higher antioxidant activity in comparison to their C7 hydroxy analogues. The obtained results confirm that the presence of hydroxyl groups in the *ortho* position affects high antioxidant activity. Among the tested compounds without a catechol moiety in the ring A (**1**–**8**), in all performed tests, luteolin (**3**) showed the highest activity. This common flavone that is extensively found in many plant species has a catechol moiety in the B-ring and the double bond at the C2–C3 position conjugated with the carbonyl group at the C-4 position, which is responsible for electron delocalization from ring B and results in significantly increased stability and, consequently, increased activity to scavenge free radicals [8,26,27]. Further analysis of the results obtained allowed us to conclude that the catechol moiety in the ring A determines equally high antioxidant activity as the catechol moiety in the ring B, which follows from the comparison of the activity of the pair of flavanones **7** (with the catechol moiety in the ring B) and **13** (with catechol moiety in the ring A) and flavones **3** and **9**, structural analogues of **7** and **13** with an unsaturated C2–C3 bond. Taking as an example the four flavonoids mentioned above (**3**, **7**, **9**, and **13**), it can be also stated that the impact of the A-ring catechol moiety on antioxidant activity is slightly stronger for flavanones. The C2–C3 double bond is generally described in the scientific literature as a structural element that increases the ability of scavenging free radicals [8,26]. However, the obtained results indicate that the presence of a double bond positively affects the antioxidant activities of flavonoids with a catechol moiety in ring B. For flavonoids with a catechol moiety in ring A, whose ring B is unsubstituted or contains a single hydroxyl group at the C-4’ position or a hydroxyl group at the position C-3' and a methoxy group at the C-4’ position, the presence of a C2–C3 double bond decreases the antioxidant activity. It is worth mentioning that some literature data indicate that *ortho*-hydroxyl substitutions, whether on the B- or A-ring, are the most important feature of the antioxidant activity of flavonoid compounds, while additional substitution seems to have no obvious effect [28,29].

The significant impact of the *ortho*-dihydroxy systems has also been observed in our research on the ability of tested flavonoids to reduce the iron ion Fe^3+^ to Fe^2+^. The compound with the highest activity proved to be 8-hydroxyluteolin (**11**), which is consistent with the observation of Arora et al., who recognized the C3’–C4’ catechol moiety in the ring B, the C2–C3 double bond, the C5-hydroxyl group, and the C7–C8 catechol moiety in the ring A as the most important structural elements [13]. According to these authors, the A-ring catechol is able to compensate the absence of a B-ring catechol and become a greater determinant of flavonoid antioxidant activity.

### 4.3. Antiproliferative Activity In Vitro

The results clearly indicate that the highest antiproliferative activity among the tested flavonoid compounds was exhibited by the flavones apigenin (**2**), luteolin (**3**), and diosmetin (**4**), while the most sensitive cell lines were MV-4-11 (human biphenotypic B myelomonocytic leukemia) and LoVo (human colon cancer). IC_50_ values for the abovementioned compounds against the MV-4-11 cell line were 8–9 μM, whereas for the LoVo cell line, they were 14–15 μM. The noteworthy feature of chrysin (**1**) is the twofold higher antiproliferative activity towards doxorubicin-resistant colon cancer cells (LoVo/DX) compared with the doxorubicin-sensitive cell line LoVo (IC_50_: 28.01 μM for LoVo and 12.98 μM for LoVo/DX). A similar relationship can be noted for pinocembrin (**5**), a structural analogue of chrysin (**1**) with a flavanone skeleton. 

The conducted studies clearly indicate that the flavonoid derivatives containing a catechol moiety at the C7–C8 position do not exhibit antiproliferative activity in vitro or it is very low. Only in the case of the MCF7 cell line, flavonoids with a catechol moiety in the ring A were more active than their corresponding nonhydroxylated analogues (**6**, **7**, and **8**). However, as already was mentioned, their antiproliferative potency was still low (IC_50_ 122–135 μM).

To avoid misleading conclusions about the unsatisfactory antiproliferative activity of compounds with the C7–C8 catechol moiety, we have undertaken research aimed at determining the stability of these flavonoids under the conditions of the antiproliferative tests performed. The research was carried out on two pairs of structural analogues: a flavone skeleton (apigenin (**2**) and 8-hydroxyapigenin (**10**)) and a flavanone skeleton (naringenin (**6**) and the mixture of 8-hydroxynaringenin (**14**) and 6-hydroxynaringenin (**15**)). Two cell lines were chosen for this study: HLMECs, which are sensitive to flavonoids with a catechol moiety, and the insensitive MCF7 cell line. The stability of the selected flavonoids at the stage of preparation of stock solutions and after addition to cell cultures or culture media without cells was examined.

The flavonoids tested (**2**, **6**, **10**, **14**, and **15**) were found stable in DMSO, and over 99% of the compound was observed after 3 h of incubation. However, the subsequent dilution of these organic solutions with a 1:1 (v/v) mixture of RPMI 1640 and Opti-MEM medium supplemented by 2 mM l-glutamine, 5% fetal bovine serum, 100 units/mL penicillin, and 100 µg/mL streptomycin resulted in the complete decomposition of 8-hydroxyapigenin (**10**), 8-hydroxynaringenin (**14**), and 6-hydroxynaringenin (**15**) within the time of incubation (3 h) and following two experiments. The analogues of these compounds lacking the hydroxyl group at the C8 position (**2**, **6**) were stable under these conditions. They were also stable during 3 h of incubation in HLMEC and MCF7 cultures (over 98%), as well as in culture media without cells (over 98%).

These observations led us to the conclusion that the cellular effect of flavonoids with a catechol moiety in the ring A, noticeable especially in relation to normal HLMECs, results from the action of the degradation products of the compounds used rather than from mother compounds or their cell metabolites. 

The problem of the instability of many flavonoids in terms of in vitro tests, which predominate in the assessment of the biological activity of compounds, is now more often addressed in the literature [30,31,32], and some relationships between the structure of polyphenols and their stability have been established [31]. These results are consistent with our observations that the pyrogallol and catechol moiety in the A-ring significantly reduce the stability of flavonoids. In this article, for the first time, this phenomenon was described for a panel of closely structurally related flavonoids, including 10 with hydroxyl groups in *ortho*-systems located in the A-ring.

## 5. Conclusions

In this work, we demonstrated that flavones and flavanones with a C7–C8 catechol moiety in the ring A are characterized as much greater antioxidants relative to their C-7 hydroxy analogues. Our observations concerning the instability of flavonoids with the catechol moiety in ring A under the conditions of commonly used tests for antiproliferative activity do not allow us to draw conclusions about the structure–antiproliferative activity relationships. However, they indicate and validate the need for stability examinations of compounds tested in cell culture assays to avoid erroneous claims, which will hopefully encourage other scientists to update the existing methodology of in vitro studies.

## Figures and Tables

**Table 1:** Inhibitory concentration 50 (IC_50_) values representing the antiproliferative activity of tested flavonoids against human cancer and normal cell lines.

Compound	IC_50_ ± SD (µM)						
	MV-4-11	MCF7	LoVo	LoVo/DX	DU 145	MCF 10A	HLMEC
**1**	15.14 ± 1.99	74.97 ± 26.08	28.01 ± 8.17	12.98 ± 1.29	ND	26.98 ± 11.96	22.85 ± 2.02
**2**	8.18 ± 2.76	22.50 ± 1.29	13.80 ± 1.38	11.58 ± 1.35	28.86 ± 8.4	35.67 ± 11.93	14.06 ± 0.91
**3**	8.73 ± 2.23	25.50 ± 6.33	13.45 ± 0.65	13.69 ± 1.65	21.94 ± 6.11	33.12 ± 10.31	9.22 ± 0.64
**4**	8.39 ± 3.53	122.73 ± 37.36	14.99 ± 2.2	13.39 ± 3.02	ND	ND	14.39 ± 1.95
**5**	99.12 ± 34.11	147.63 ± 6.57	116.10 ± 10.63	67.04 ± 15.81	118.60 ± 22.45	125.19 ± 5.77	96.59 ± 7.23
**6**	146.19 ± 19.09	215.35 ± 32.28	120.84 ± 4.3	128.67 ± 9.74	241.65 ± 15.61	185.16 ± 23.19	113.98 ± 18.55
**7**	ND	150.70 ± 9.31	100.68 ± 10.86	99.22 ± 12.46	120.66 ± 18.45	146.61 ± 36.21	25.81 ± 6.71
**8**	140.47 ± 12.93	167.33 ± 13.12	103.81 ± 5.55	77.25 ± 19.39	214.54 ± 32.09	206.97 ± 23.42	98.29 ± 7.62
**9**	ND	ND	ND	ND	ND	ND	119.60 ± 2.24
**10**	ND	ND	235.29 ± 57.07	ND	ND	ND	122.21 ± 14.38
**11**	ND	ND	237.76 ± 35.36	ND	ND	ND	105.98 ± 1.72
**12**	ND	ND	223.65 ± 15.29	ND	ND	ND	107.70 ± 15.4
**13**	ND	ND	ND	ND	ND	ND	148.50 ± 13.38
14 and 15 *	ND	121.80 ± 4.11	ND	ND	ND	ND	110.22 ± 0.18
16 and 17 **	ND	115.53 ± 7.1	179.49 ± 31.97	ND	ND	202.93 ± 93.02	90.68 ± 18.85
**18**	ND	135.45 ± 20.25	ND	ND	ND	ND	97.06 ± 5.15
**Cisplatin**	1.60 ± 0.33	7.50 ± 1.32	5.87 ± 1.72	3.23 ± 0.77	1.47 ± 0.12	10.63 ± 1.17	1.07 ± 0.05

SD—standard deviation; ND—not determined in the range of tested concentrations (100; 10; 1; 0.1 µg/mL); * ratio 19:1 (by HPLC); ** ratio 1:1.3 (by HPLC).

## Supplementary Materials

The following are available online at https://www.mdpi.com/2076-3921/8/7/210/s1, Figure S1: ^1^H-NMR (600 MHz, DMSO-*d_6_*) spectrum of 8-hydroxydiosmetin (**12**), Figure S2: ^13^C-NMR (150 MHz, DMSO-*d_6_*) spectrum of 8-hydroxydiosmetin (**12**), Figure S3: ^1^H-NMR (600 MHz, DMSO-*d_6_*) spectrum8-hydroxypinocembrin (**13**), Figure S4: ^13^C-NMR (150 MHz, DMSO-*d_6_*) spectrum 8-hydroxypinocembrin (**13**), Figure S5: ^1^H-NMR (600 MHz, DMSO-*d_6_*) spectrum of the mixture of 8-hydroxyeriodictyol and 6-hydroxyeriodictyol (**16**, **17**), Figure S6: ^13^C-NMR (150 MHz, DMSO-*d_6_*) spectrum of the mixture of 8-hydroxyeriodictyol and 6-hydroxyeriodictyol (**16**, **17**), Table S1: Antioxidant activity of tested flavonoids (**1**–**18**) determined in separate tests: ABTS, DPPH, and FRAP.

## Sample Availability

Samples of all compounds are available from the authors for possible research projects in cooperation.
