# Supplementary material for: Structure–Antioxidant–Antiproliferative Activity Relationships of Natural C7 and C7–C8 Hydroxylated Flavones and Flavanones
Source: Antioxidants (Basel). 2019 Jul 7;8(7):210. doi: 10.3390/antiox8070210 (PMC6680932; doi:10.3390/antiox8070210)
Supplement: Supplementary file 1 [file antioxidants-08-00210-s001.pdf]

Supplementary Materials:

Structure - antioxidant - antiproliferative activity relationships of natural C7 and C7-C8 hydroxylated flavones and flavanones

Sandra Sordon[a]*, Jarosław Popłoński[a], Magdalena Milczarek[b], Martyna Stachowicz[b], Tomasz Tronina[a], Alicja Z. Kucharska[c], Joanna Wietrzyk[b], Ewa Huszcza[a].

a Department of Chemistry, Wrocław University of Environmental and Life Sciences, Norwida 25, 50-375 Wrocław, Poland

b Hirszfeld Institute of Immunology and Experimental Therapy, Polish Academy of Sciences, Weigla 12, 53-114 Wrocław, Poland

c Department of Fruit, Vegetable and Plant Nutraceutical Technology, Wrocław University of Environmental and Life Sciences, Chełmońskiego 37, 51-630 Wrocław, Poland

*Correspondence: sandra.sordon@upwr.edu.pl

List of contents:

1. NMR spectra of 8-hydroxydiosmetin (**12**).
   Figure S1: ^{1}H-NMR (600 MHz, DMSO-d_6) spectrum.
   Figure S2: ^{13}C-NMR (150 MHz, DMSO-d_6) spectrum.

2. NMR spectra of 8-hydroxypinocembrin (**13**).
   Figure S3: ^{1}H-NMR (600 MHz, DMSO-d_6) spectrum.
   Figure S4: ^{13}C-NMR (150 MHz, DMSO-d_6) spectrum.

3. NMR spectra of the mixture of 8-hydroxyeriodictyol and 6-hydroxyeriodictyol (**16**, **17**).
   Figure S5: ^{1}H-NMR (600 MHz, DMSO-d_6) spectrum.
   Figure S6: ^{13}C-NMR (150 MHz, DMSO-d_6) spectrum.

4. Table S1: antioxidant activity of tested flavonoids (**1-18**) determined in separate tests: ABTS, DPPH and FRAP.

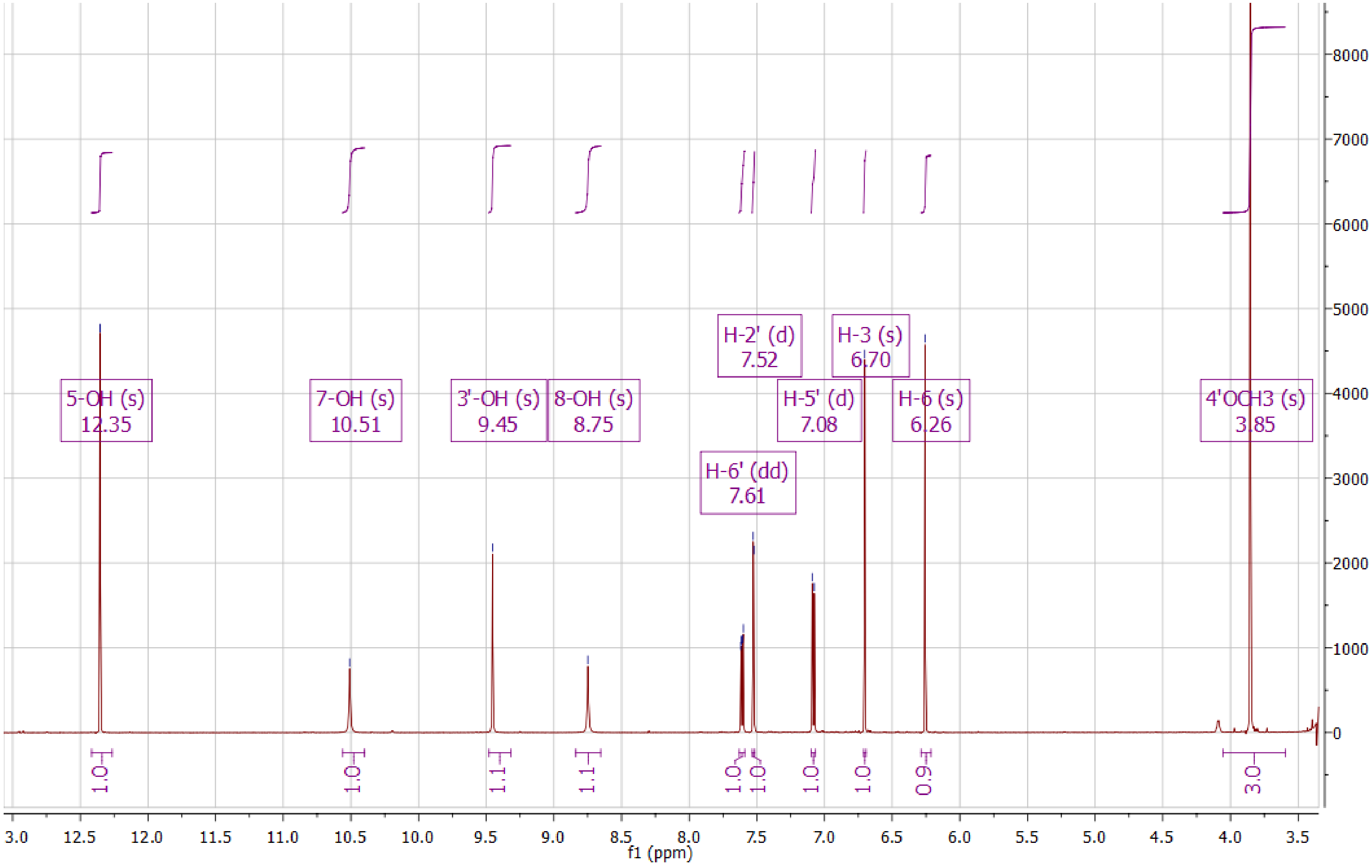

Figure S1. ^{1}H-NMR (600 MHz, DMSO-d_6) spectrum of 8-hydroxydiosmetin (**12**).

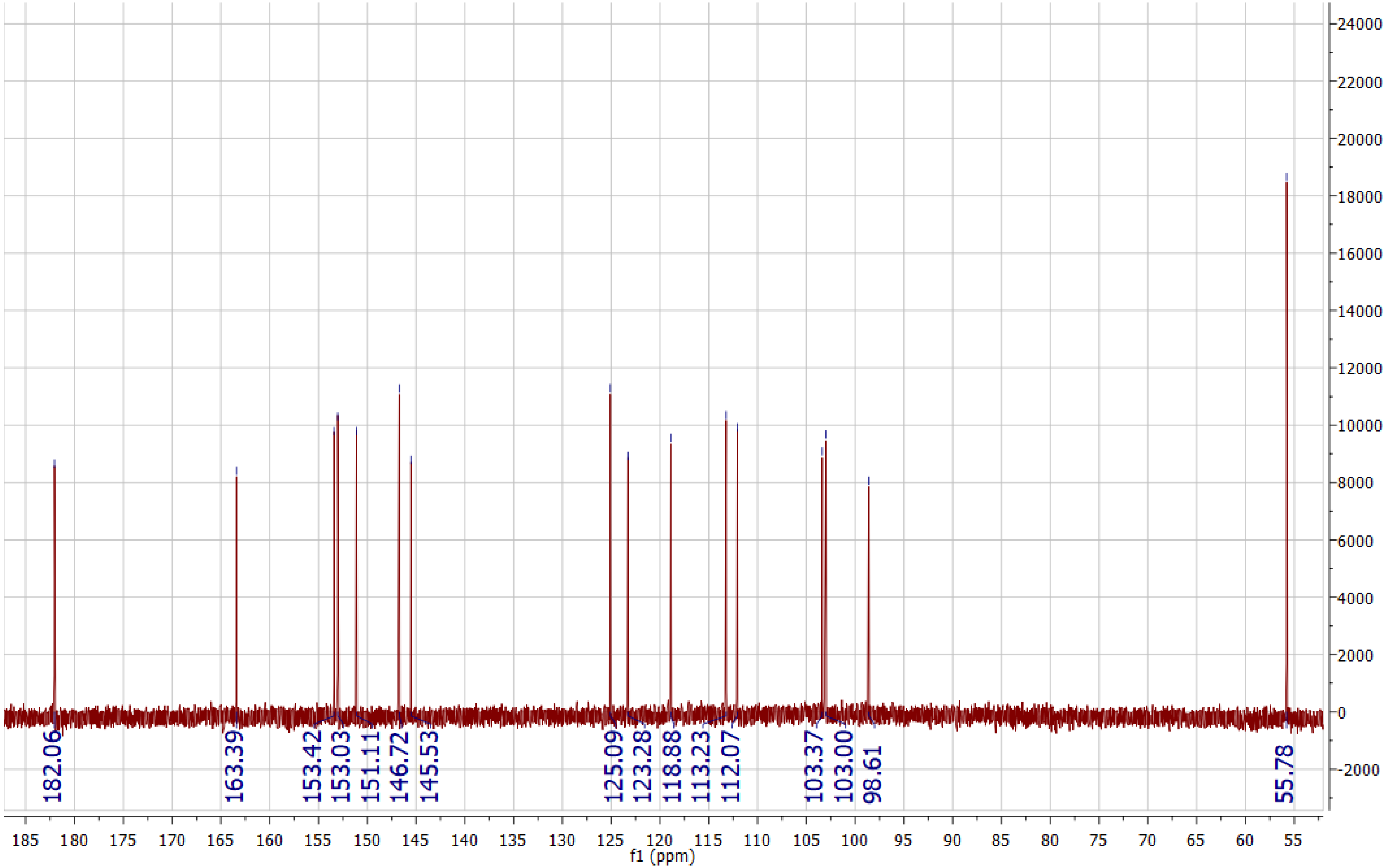

Figure S2. ^{13}C-NMR (150 MHz, DMSO-d_6) spectrum of 8-hydroxydiosmetin (**12**).

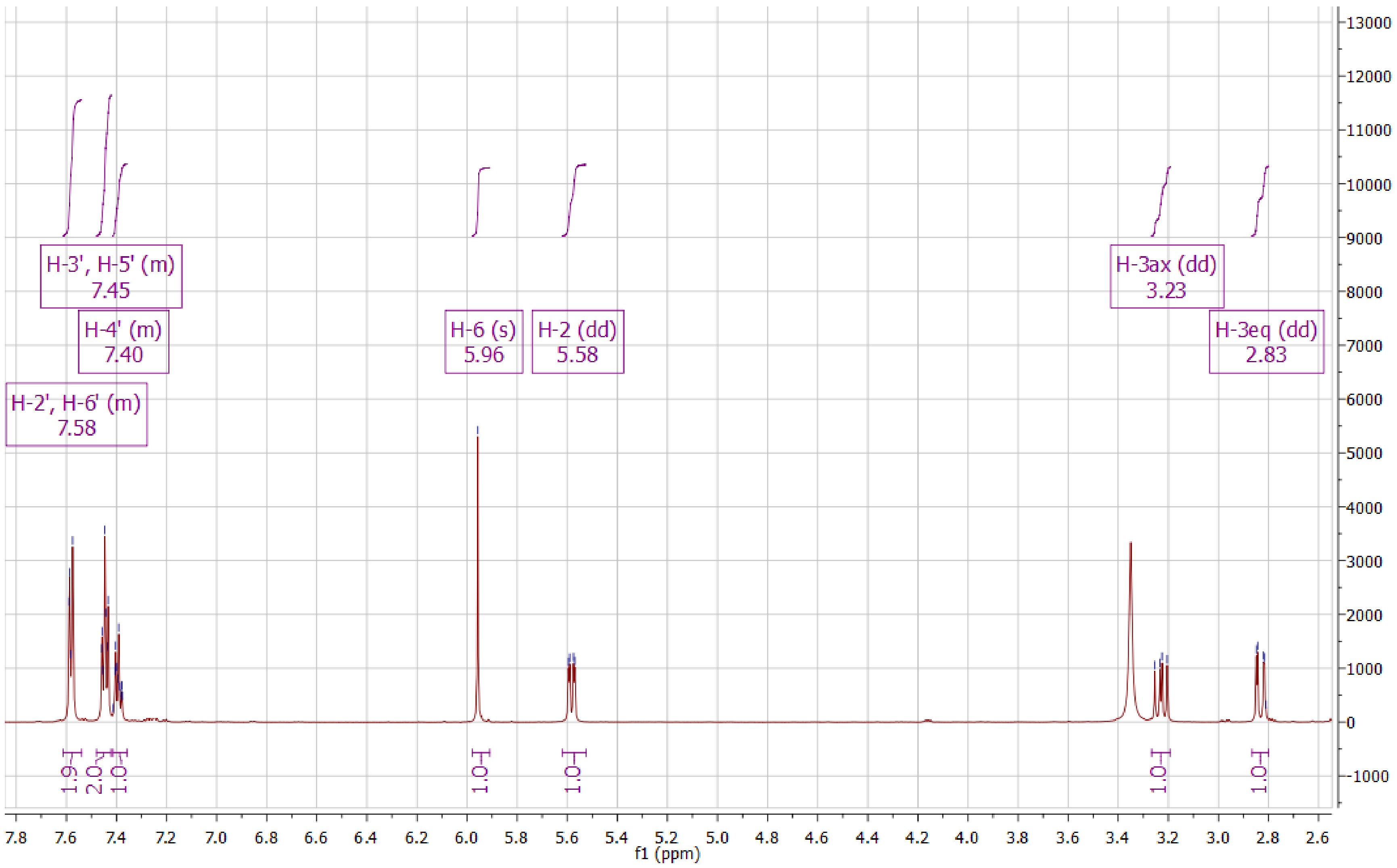

Figure S3. ^{1}H-NMR (600 MHz, DMSO-d_6) spectrum of 8-hydroxypinocembrin (**13**).

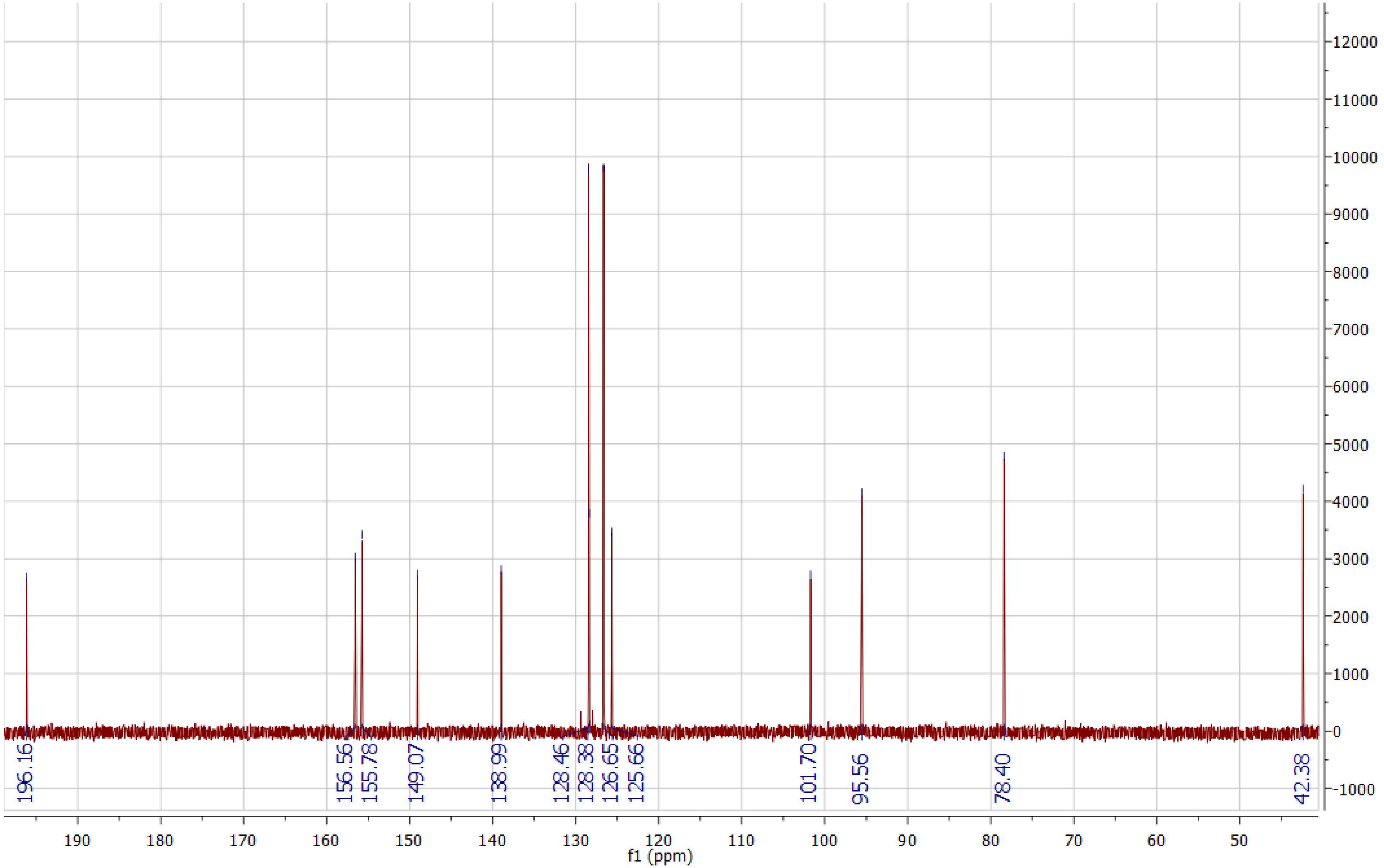

Figure S4. ^{13}C-NMR (150 MHz, DMSO-d_6) spectrum of 8-hydroxypinocembrin (**13**).

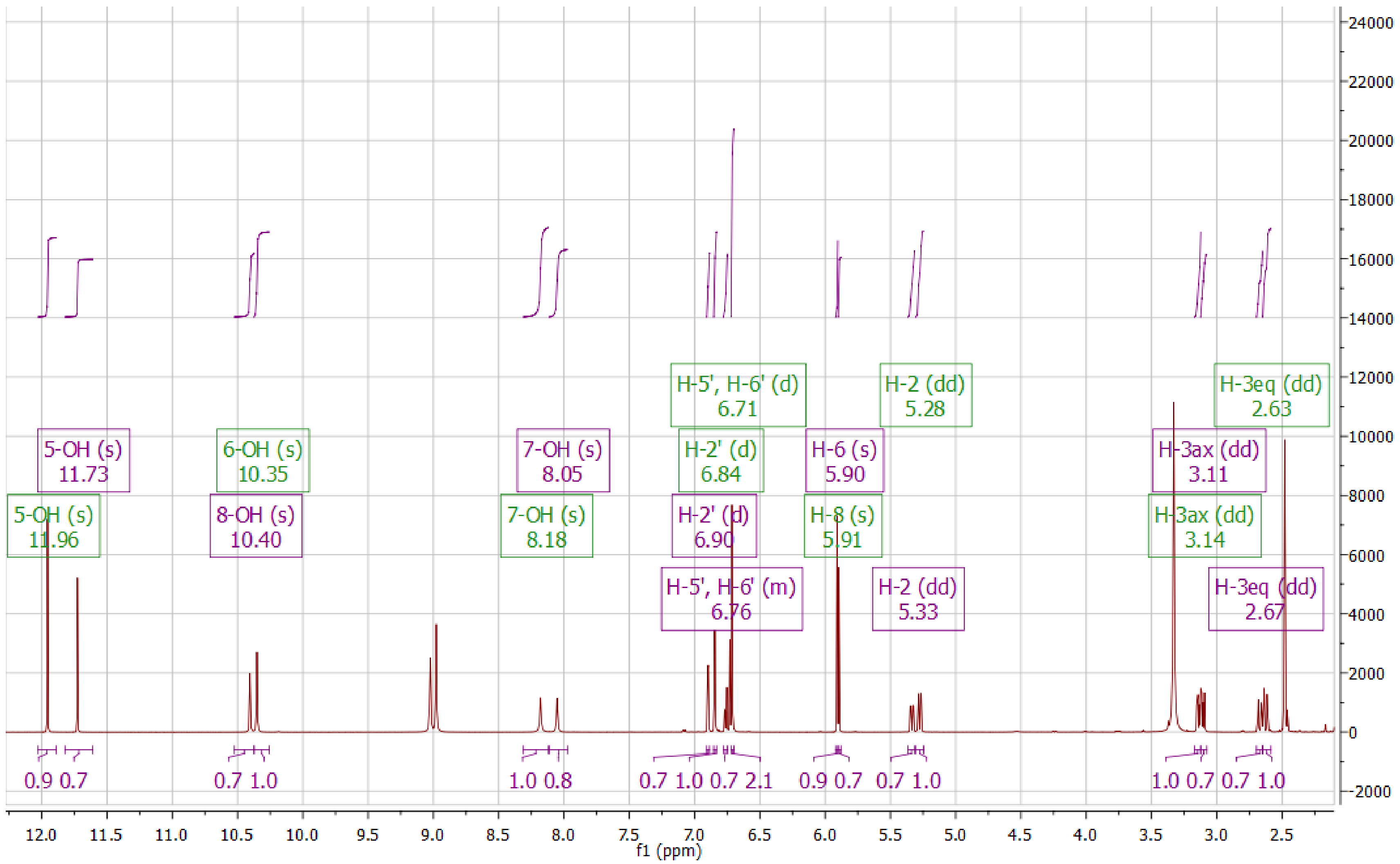

Figure S5. ^{1}H-NMR (600 MHz, DMSO-d_6) spectrum of 8-hydroxyeriodictyol (**16**) (violet) and 6-hydroxyeriodictyol (**17**) (green).

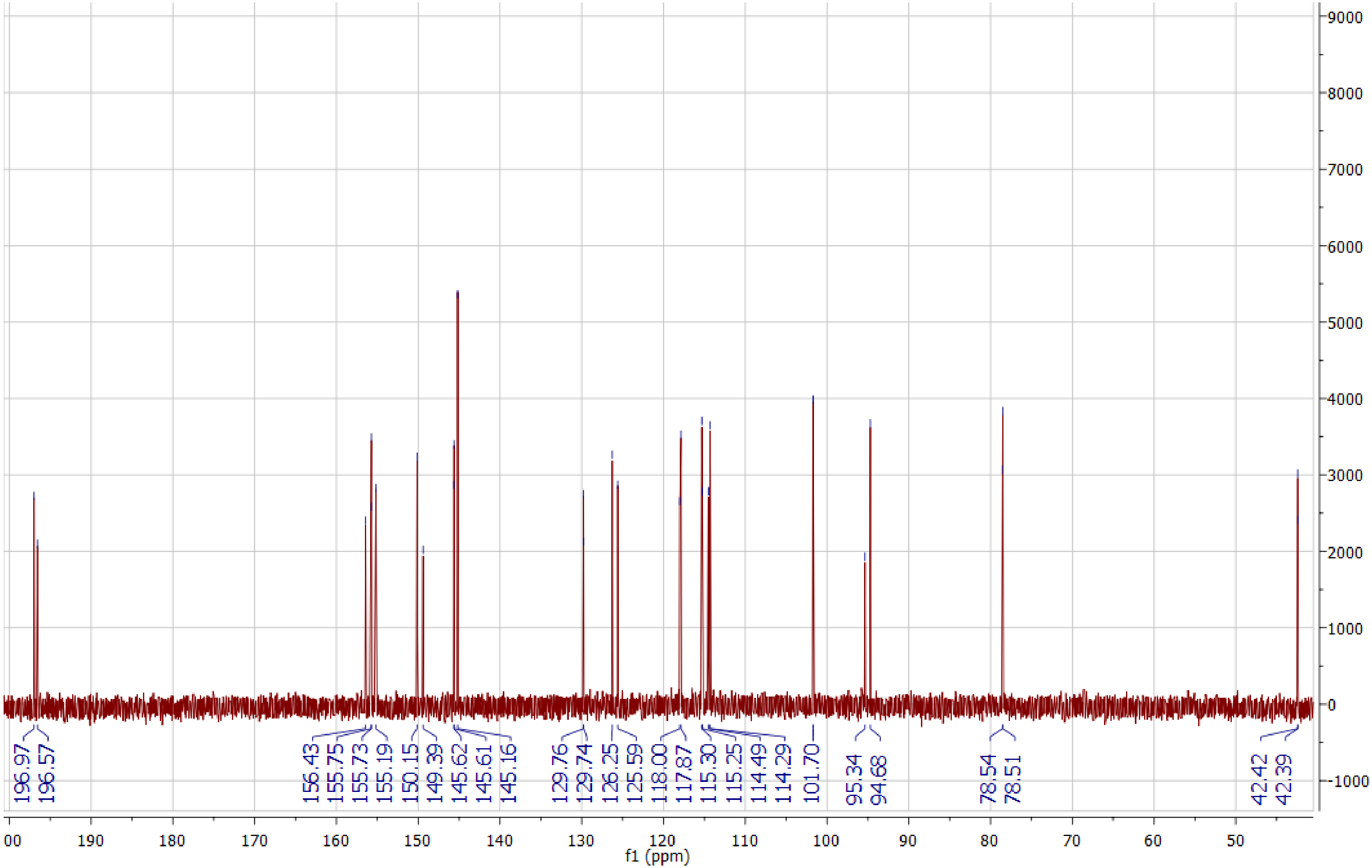

Figure S6. ^{13}C-NMR (150 MHz, DMSO-d_6) spectrum of 8-hydroxyeriodictyol and 6-hydroxyeriodictyol (**16**, **17**).

Table S1. Antioxidant activity of tested flavonoids.[a]

**Compound**	**TEAC$_{ABTS}$**	**TEAC$_{DPPH}$**	**TEAC$_{FRAP}$**
**1**	0.084 ± 0.011	0.004 ± 0.001	ND
**2**	0.250 ± 0.014	0.005 ± 0.002	ND
**3**	16.645 ± 0.897	10.50 ± 0.117	17.686 ± 0.327
**4**	1.712 ± 0.041	0.008 ± 0.001	ND
**5**	0.165 ± 0.009	0.005 ± 0.001	ND
**6**	0.347 ± 0.020	0.017 ± 0.002	ND
**7**	13.988 ± 0.251	9.582 ± 0.315	10.619 ± 0.319
**8**	13.254 ± 0.123	0.098 ± 0.002	4.025 ± 0.175
**9**	14.173 ± 1.591	8.038 ± 0.061	8.900 ± 0.582
**10**	10.184 ± 1.768	5.560 ± 0.121	7.824 ± 0.657
**11**	34.450 ± 1.016	13.208 ± 0.080	36.895 ± 0.432
**12**	13.405 ± 0.826	8.496 ± 0.040	11.340 ± 0.203
**13**	20.763 ± 0.939	9.164 ± 0.097	12.861 ± 0.313
**14** and **15***	17.370 ± 0.989	9.098 ± 0.045	8.686 ± 0.093
**16** and **17****	29.243 ± 1.362	14.649 ± 0.182	22.367 ± 1.801
**18**	31.187 ± 1.353	14.150 ± 0.229	25.260 ± 0.909

[a] Results are expressed as the equivalent of µmol trolox (TEAC$_{ABTS}$, TEAC$_{DPPH}$, TEAC$_{FRAP}$) per µmol of tested compound; SD – standard deviation; ND – not determined in the range of concentrations tested (50 µg/mL - 10 mg/mL); *ratio 19:1 (by HPLC); **ratio 1:1.3 (by HPLC).
